# Supplementary material for: Bridging the Gap: Two Decades of Childhood Vaccination Coverage and Equity in Cambodia and the Philippines (2000–2022)
Source: Vaccines (Basel). 2025 Aug 27;13(9):907. doi: 10.3390/vaccines13090907 (PMC12474049; doi:10.3390/vaccines13090907)
Supplement: Supplementary file 1 [file vaccines-13-00907-s001.zip › vaccines-3828206-supplementary.pdf]

Table S1: Urban-rural disparities in vaccination coverage on schedule, 2000–2022

Country	Year	BCG	DTP	OPV	Hepb at birth	PCV	Measles
Cambodia	2000	8.4	9.3	8.9	-	-	-
	2005	1.6	3.6	5.0	-	-	-
	2010	9.1	12	12.6	-	-	-
	2014	4.4	10.9	9.7	-	-	-
	2021–22	1.7	6.0	4.8	2.0	6.0	5.7
Philippines	2003	7.9	8.3	6.7	-	-	-
	2008	8.8	5.1	4.2	-	-	-
	2013	5.6	6.1	5.2	-	-	-
	2017	5.2	5.0	3.6	11.5	-5.4	5.1
	2022	6.9	4.5	2.5	9.9	4.0	2.6

Table S2: Wealth-related disparities in vaccination coverage on schedule, 2000–2022

Country	Year	BCG	DTP	OPV	Hepb at birth	PCV	Measles
Cambodia	2005	18.2 (95% CI: 8.6 to 27.7)	33.1 (95% CI: 20.2 to 46.0)	30.4 (95% CI: 17.3 to 43.5)	-	-	-
	2010	19.6 (95% CI: 10.9 to 28.3)	31.7 (95% CI: 20.1 to 43.3)	30.4 (95% CI: 18.7 to 42.1)	-	-	-
	2014	15.6 (95% CI: 7.2 to 24.1)	32.1 (95% CI: 20.2 to 43.9)	29.3 (95% CI: 17.2 to 41.5)	-	-	-
	2021–22	9.2 (95% CI: 1.9 to 16.5)	24.4 (95% CI: 13.2 to 35.6)	21.2 (95% CI: 10.3 to 32.0)	10.7 (95% CI: 3.4 to 18.0)	22.8 (95% CI: 11.6 to 34.0)	25.2 (95% CI: 0.4 to 50.1)
Philippines	2003	25.4 (95% CI: 14.4 to 36.4)	31.3 (95% CI: 17.3 to 45.3)	28.8 (95% CI: 14.5 to 43.0)	-	-	-
	2008	28.2 (95% CI: 17.3 to 39.1)	30.9 (95% CI: 17.2 to 44.6)	30.5 (95% CI: 16.7 to 44.3)	-	-	-
	2013	24.2 (95% CI: 14.5 to 33.9)	27.2 (95% CI: 14.1 to 40.3)	25.9 (95% CI: 12.5 to 39.3)	-	-	-
	2017	23.6 (95% CI: 15.0 to 32.2)	25.5 (95% CI: 14.0 to 37.1)	20.3 (95% CI: 8.3 to 32.1)	38.5 (95% CI: 28.8 to 48.2)	7.5 (95% CI: -5.8 to 20.8)	19.6 (95% CI: 1.7 to 37.5)
	2022	32.1 (95% CI: 22.1 to 42.0)	39.8 (95% CI: 28.0 to 51.6)	36.1 (95% CI: 23.7 to 48.4)	42.9 (95% CI: 32.7 to 53.2)	38.7 (95% CI: 26.0 to 51.3)	37.1 (95% CI: 19.5 to 54.8)

Table S3: Factors associated with not receiving BCG and Hep B vaccines at birth in Cambodia and the Philippines, 2003–2022

Variables	Adjusted[1] OR (95%CI)	p-value
Cambodia		
**BCG**		
**Year**		
2005	Ref.	Ref.
2010	0.80 (0.67, 0.96)	0.02
2014	0.69 (0.57, 0.85)	<0.001
2021	0.74 (0.61, 0.90)	0.003
**Maternal age**		
30+	Ref.	Ref.
20–29	1.02 (0.87, 1.20)	0.7
<19	1.39 (1.05, 1.85)	0.02
**Residence**		
Urban	Ref.	Ref.
Rural	1.00 (0.83, 1.21)	1.0
**Education**		
Secondary and higher	Ref.	Ref.
Primary	0.93 (0.65, 1.35)	0.7
Incomplete primary	1.26 (0.88, 1.84)	0.2
No education	2.80 (1.93, 4.14)	<0.001
**Wealth index**		
Richest	Ref.	Ref.
Richer	1.06 (0.79, 1.44)	0.7
Middle	1.51 (1.12, 2.03)	0.007
Poorer	1.90 (1.42, 2.55)	<0.001
Poorest	2.34 (1.76, 3.13)	<0.001
**Employment**		
Work	Ref.	Ref.
Did not work	0.92 (0.78, 1.09)	0.4
**Parity**		
1	Ref.	Ref.
2	1.04 (0.86, 1.25)	0.7
3+	1.33 (1.10, 1.62)	0.004
**Hep B (at birth)**		
**Maternal age**		
30+	Ref.	Ref.
20–29	1.01 (0.72, 1.42)	0.9
<19	2.55 (1.53, 4.25)	<0.001

[1] Adjusting for all explanatory variables

**Residence**		
Urban	Ref.	Ref.
Rural	0.96 (0.69, 1.33)	0.8
**Education**		
Secondary and higher	Ref.	Ref.
Primary	1.31 (0.79, 2.25)	0.3
Incomplete primary	1.27 (0.74, 2.27)	0.4
No education	2.78 (1.55, 5.15)	<0.001
**Wealth index**		
Richest	Ref.	Ref.
Richer	1.03 (0.61, 1.79)	0.9
Middle	0.91 (0.52, 1.62)	0.7
Poorer	0.91 (0.51, 1.65)	0.8
Poorest	1.85 (1.09, 3.23)	0.03
**Employment**		
Work	Ref.	Ref.
Did not work	0.92 (0.70, 1.21)	0.6
**Parity**		
1	Ref.	Ref.
2	0.88 (0.62, 1.27)	0.5
3+	1.04 (0.69, 1.58)	0.9
Philippines		
**BCG**		
**Year**		
2003	Ref.	Ref.
2008	0.82 (0.70, 0.97)	0.02
2013	0.49 (0.41, 0.59)	<0.001
2017	0.78 (0.68, 0.91)	0.001
2022	1.01 (0.87, 1.17)	0.9
**Maternal age**		
30+	Ref.	Ref.
20–29	1.10 (0.98, 1.24)	0.09
<19	1.10 (0.88, 1.36)	0.4
**Residence**		
Urban	Ref.	Ref.
Rural	1.16 (1.03, 1.30)	0.01
**Education**		
Secondary and higher	Ref.	Ref.
Primary	1.24 (1.10, 1.41)	<0.001
Incomplete primary	2.09 (1.79, 2.45)	<0.001
No education	5.19 (3.93, 6.86)	<0.001
**Wealth index**		

Richest	Ref.	Ref.
Richer	1.38 (1.05, 1.81)	0.02
Middle	1.57 (1.22, 2.04)	<0.001
Poorer	1.82 (1.42, 2.36)	<0.001
Poorest	3.25 (2.54, 4.21)	<0.001
**Employment**		
Work	Ref.	Ref.
Did not work	1.15 (1.03, 1.28)	0.01
**Parity**		
1	Ref.	Ref.
2	1.00 (0.85, 1.16)	0.9
3+	1.26 (1.09, 1.47)	0.002
**Hep B (at birth)**		
**Maternal age**		
30+	Ref.	Ref.
20–29	1.26 (1.10, 1.44)	<0.001
<19	1.37 (1.06, 1.76)	0.02
**Residence**		
Urban	Ref.	Ref.
Rural	1.26 (1.10, 1.44)	<0.001
**Education**		
Secondary and higher	Ref.	Ref.
Primary	1.27 (1.10, 1.46)	0.001
Incomplete primary	2.30 (1.88, 2.81)	<0.001
No education	5.18 (3.35, 8.16)	<0.001
**Wealth index**		
Richest	Ref.	Ref.
Richer	1.32 (0.97, 1.79)	0.08
Middle	1.62 (1.23, 2.17)	<0.001
Poorer	1.86 (1.42, 2.47)	<0.001
Poorest	3.25 (2.48, 4.31)	<0.001
**Employment**		
Work	Ref.	Ref.
Did not work	1.09 (0.96, 1.24)	0.2
**Parity**		
1	Ref.	Ref.
2	1.21 (1.02, 1.43)	0.03
3+	1.49 (1.26, 1.77)	<0.001

Table S4: Factors associated with incomplete and 0 dose of measles vaccine, DTP, OPV and PCV in Cambodia and the Philippines, 2000–2022

	Incomplete		0 dose	
**Variables**	**Adjusted[1] OR (95%CI)**	**p-value**	**Adjusted[2] OR (95%CI)**	**p-value**
Cambodia				
**Measles**				
**Maternal age**				
30+	Ref.	Ref.	Ref.	Ref.
20–29	1.26 (0.82, 1.95)	0.3	1.51 (0.92, 2.48)	0.1
<19	1.09 (0.46, 2.60)	0.8	1.83 (0.75, 4.44)	0.2
**Residence**				
Urban	Ref.	Ref.	Ref.	Ref.
Rural	0.87 (0.57, 1.33)	0.5	1.08 (0.67, 1.72)	0.8
**Education**				
Secondary and higher	Ref.	Ref.	Ref.	Ref.
Primary	1.42 (0.76, 2.65)	0.3	1.49 (0.72, 3.08)	0.3
Incomplete primary	1.85 (0.95, 3.60)	0.07	1.56 (0.71, 3.42)	0.3
No education	2.85 (1.26, 6.43)	0.01	4.16 (1.71, 10.11)	0.002
**Wealth index**				
Richest	Ref.	Ref.	Ref.	Ref.
Richer	1.29 (0.67, 2.48)	0.4	1.29 (0.61, 2.70)	0.5
Middle	1.39 (0.69, 2.78)	0.4	1.44 (0.66, 3.14)	0.4
Poorer	1.59 (0.80, 3.18)	0.2	0.98 (0.43, 2.21)	1.0
Poorest	1.72 (0.86, 3.44)	0.1	1.62 (0.75, 3.52)	0.2
**Employment**				
Work	Ref.	Ref.	Ref.	Ref.
Did not work	0.69 (0.46, 1.04)	0.08	0.97 (0.63, 1.49)	0.9
**Parity**				
1	Ref.	Ref.	Ref.	Ref.
2	1.37 (0.86, 2.19)	0.2	1.01 (0.59, 1.74)	1.0
3+	1.63 (0.93, 2.85)	0.09	2.26 (1.23, 4.16)	0.009
**OPV**				

[1] Adjusting for all explanatory variables
[2] Adjusting for all explanatory variables

**Year**				
2000	Ref.	Ref.	Ref.	Ref.
2005	0.75 (0.67, 0.85)	<0.01	0.57 (0.48, 0.67)	<0.01
2010	0.58 (0.52, 0.65)	<0.01	0.52 (0.44, 0.60)	<0.01
2014	0.72 (0.64, 0.80)	<0.01	0.47 (0.40, 0.56)	<0.01
2021	0.44 (0.39, 0.49)	<0.01	0.40 (0.34, 0.47)	<0.01
**Maternal age**				
30+	Ref.	Ref.	Ref.	Ref.
20–29	1.05 (0.92, 1.20)	0.5	1.28 (1.07, 1.53)	0.007
<19	1.34 (1.06, 1.70)	0.01	1.98 (1.44, 2.72)	<0.001
**Residence**				
Urban	Ref.	Ref.	Ref.	Ref.
Rural	0.94 (0.81, 1.10)	0.5	1.08 (0.87, 1.35)	0.5
**Education**				
Secondary and higher	Ref.	Ref.	Ref.	Ref.
Primary	1.52 (1.14, 2.03)	0.005	0.91 (0.59, 1.40)	0.7
Incomplete primary	2.35 (1.74, 3.16)	<0.001	1.68 (1.08, 2.59)	0.02
No education	3.27 (2.38, 4.48)	<0.01	4.06 (2.59, 6.36)	<0.01
**Wealth index**				
Richest	Ref.	Ref.	Ref.	Ref.
Richer	0.96 (0.78, 1.19)	0.7	1.13 (0.81, 1.59)	0.5
Middle	1.35 (1.09, 1.66)	0.006	1.45 (1.03, 2.03)	0.03
Poorer	1.44 (1.17, 1.79)	<0.001	1.69 (1.21, 2.37)	0.002
Poorest	1.57 (1.27, 1.95)	<0.001	2.47 (1.78, 3.43)	<0.001
**Employment**				
Work	Ref.	Ref.	Ref.	Ref.
Did not work	1.02 (0.89, 1.17)	0.8	0.92 (0.76, 1.12)	0.4
**Parity**				
1	Ref.	Ref.	Ref.	Ref.
2	1.02 (0.88, 1.18)	0.8	1.03 (0.83, 1.27)	0.8
3+	1.21 (1.04, 1.42)	0.02	1.61 (1.30, 2.00)	<0.001
**DTP**				
**Year**				
2000	Ref.	Ref.	Ref.	Ref.
2005	0.72 (0.63, 0.81)	<0.01	0.56 (0.48, 0.65)	<0.01
2010	0.57 (0.51, 0.64)	<0.01	0.51 (0.44, 0.58)	<0.01

2014	0.64 (0.57, 0.72)	<0.01	0.48 (0.41, 0.56)	<0.01
2021	0.45 (0.40, 0.51)	<0.01	0.55 (0.48, 0.63)	<0.01
**Maternal age**				
30+	Ref.	Ref.	Ref.	Ref.
20–29	1.05 (0.92, 1.20)	0.5	1.24 (1.05, 1.47)	0.01
<19	1.39 (1.09, 1.77)	0.007	1.86 (1.38, 2.50)	<0.001
**Residence**				
Urban	Ref.	Ref.	Ref.	Ref.
Rural	0.89 (0.77, 1.04)	0.2	1.13 (0.92, 1.38)	0.3
**Education**				
Secondary and higher	Ref.	Ref.	Ref.	Ref.
Primary	1.58 (1.16, 2.14)	0.004	0.84 (0.58, 1.21)	0.3
Incomplete primary	2.26 (1.65, 3.10)	<0.001	1.49 (1.02, 2.16)	0.04
No education	3.38 (2.42, 4.71)	<0.01	3.54 (2.40, 5.22)	<0.01
**Wealth index**				
Richest	Ref.	Ref.	Ref.	Ref.
Richer	1.05 (0.84, 1.30)	0.7	1.07 (0.78, 1.47)	0.7
Middle	1.42 (1.14, 1.77)	0.002	1.37 (1.00, 1.87)	0.05
Poorer	1.63 (1.31, 2.03)	<0.001	1.66 (1.22, 2.26)	0.001
Poorer	1.79 (1.43, 2.23)	<0.01	2.49 (1.84, 3.37)	<0.01
**Employment**				
Work	Ref.	Ref.	Ref.	Ref.
Did not work	1.39 (0.95, 1.25)	0.2	0.87 (0.73, 1.04)	0.1
**Parity**				
1	Ref.	Ref.	Ref.	Ref.
2	1.11 (0.95, 1.29)	0.2	1.01 (0.83, 1.23)	0.9
3+	1.31 (1.11, 1.54)	0.001	1.43 (1.17, 1.75)	<0.001
**PCV**				
**Maternal age**				
30+	Ref.	Ref.	Ref.	Ref.
20–29	1.05 (0.79, 1.38)	0.7	1.37 (0.97, 1.93)	0.07
<19	1.57 (0.95, 2.60)	0.08	2.86 (1.62, 5.06)	<0.001
**Residence**				
Urban	Ref.	Ref.	Ref.	Ref.
Rural	0.95 (0.72, 1.26)	0.7	1.13 (0.79, 1.60)	0.5
**Education**				

Secondary and higher	Ref.	Ref.	Ref.	Ref.
Primary	1.82 (1.12, 2.96)	0.02	1.03 (0.60, 1.76)	0.9
Incomplete primary	2.43 (1.45, 4.04)	<0.001	1.12 (0.63, 1.98)	0.7
No education	2.99 (1.69, 5.28)	<0.001	2.15 (1.16, 4.00)	0.02
**Wealth index**				
Richest	Ref.	Ref.	Ref.	Ref.
Richer	0.77 (0.48, 1.24)	0.3	0.96 (0.53, 1.75)	0.9
Middle	1.40 (0.88, 2.20)	0.2	1.31 (0.72, 2.38)	0.4
Poorer	1.23 (0.76, 1.97)	0.4	1.09 (0.58, 2.03)	0.8
Poorer	1.37 (0.86, 2.20)	0.2	2.11 (1.17, 3.80)	0.01
**Employment**				
Work	Ref.	Ref.	Ref.	Ref.
Did not work	0.91 (0.71, 1.16)	0.4	1.10 (0.83, 1.46)	0.5
**Parity**				
1	Ref.	Ref.	Ref.	Ref.
2	1.20 (0.88, 1.64)	0.3	1.14 (0.78, 1.68)	0.5
3+	1.40 (0.98, 2.01)	0.07	1.81 (1.17, 2.80)	0.007
Philippines				
**Measles**				
**Maternal age**				
30+	Ref.	Ref.	Ref.	Ref.
20–29	1.15 (0.96, 1.38)	0.1	1.31 (1.14, 1.50)	<0.001
<19	1.66 (1.21, 2.28)	0.001	1.32 (1.02, 1.71)	0.04
**Residence**				
Urban	Ref.	Ref.	Ref.	Ref.
Rural	0.99 (0.84, 1.18)	0.9	0.77 (0.68, 0.88)	<0.001
**Education**				
Secondary and higher	Ref.	Ref.	Ref.	Ref.
Primary	1.12 (0.93, 1.35)	0.2	1.15 (0.99, 1.33)	0.07
Incomplete primary	1.61 (1.17, 2.20)	0.003	2.43 (1.92, 3.08)	<0.001
No education	0.76 (0.28, 2.08)	0.6	4.40 (2.59, 7.50)	<0.001
**Wealth index**				
Richest	Ref.	Ref.	Ref.	Ref.
Richer	0.98 (0.72, 1.33)	0.9	1.35 (1.06, 1.72)	0.02
Middle	0.93 (0.70, 1.25)	0.6	1.16 (0.92, 1.47)	0.2
Poorer	0.95 (0.71, 1.27)	0.7	1.17 (0.93, 1.48)	0.2

Poorest	1.16 (0.87, 1.56)	0.3	1.62 (1.28, 2.05)	<0.001
**Employment**				
Work	Ref.	Ref.	Ref.	Ref.
Did not work	1.19 (1.01, 1.39)	0.04	1.30 (1.15, 1.48)	<0.001
**Parity**				
1	Ref.	Ref.	Ref.	Ref.
2	1.05 (0.85, 1.30)	0.7	1.14 (0.97, 1.34)	0.1
3+	1.17 (0.94, 1.46)	0.2	1.26 (1.06, 1.49)	0.008
**OPV**				
**Year**				
2003	Ref.	Ref.	Ref.	Ref.
2008	0.78 (0.66, 0.91)	0.002	0.77 (0.63, 0.93)	0.007
2013	0.85 (0.73, 0.99)	0.03	0.76 (0.63, 0.93)	0.006
2017	0.65 (0.56, 0.76)	<0.001	1.20 (1.02, 1.41)	0.03
2022	0.81 (0.69, 0.94)	0.007	1.33 (1.12, 1.58)	0.001
**Maternal age**				
30+	Ref.	Ref.	Ref.	Ref.
20–29	1.07 (0.95, 1.20)	0.2	1.14 (1.01, 1.30)	0.04
<19	1.22 (0.99, 1.51)	0.06	1.17 (0.92, 1.49)	0.2
**Residence**				
Urban	Ref.	Ref.	Ref.	Ref.
Rural	0.86 (0.77, 0.96)	0.008	0.99 (0.87, 1.12)	0.9
**Education**				
Secondary and higher	Ref.	Ref.	Ref.	Ref.
Primary	1.18 (1.04, 1.33)	0.009	1.28 (1.12, 1.47)	<0.001
Incomplete primary	1.57 (1.31, 1.87)	<0.001	2.53 (2.13, 3.01)	<0.001
No education	1.66 (1.07, 2.59)	0.02	6.65 (4.82, 9.17)	<0.001
**Wealth index**				
Richest	Ref.	Ref.	Ref.	Ref.
Richer	1.19 (0.98, 1.45)	0.08	1.43 (1.08, 1.90)	0.01
Middle	1.06 (0.87, 1.29)	0.5	1.54 (1.17, 2.02)	0.002
Poorer	1.44 (1.19, 1.74)	<0.001	1.86 (1.43, 2.43)	<0.001
Poorest	1.46 (1.19, 1.78)	<0.001	3.32 (2.54, 4.32)	<0.001
**Employment**				
Work	Ref.	Ref.	Ref.	Ref.
Did not work	1.03 (0.93, 1.14)	0.6	1.13 (1.01, 1.27)	0.03

**Parity**				
1	Ref.	Ref.	Ref.	Ref.
2	1.12 (0.97, 1.30)	0.1	1.09 (0.93, 1.29)	0.3
3+	1.20 (1.03, 1.38)	0.02	1.33 (1.12, 1.56)	<0.001
**DTP**				
**Year**				
2003	Ref.	Ref.	Ref.	Ref.
2008	0.80 (0.68, 0.94)	0.006	0.72 (0.59, 0.88)	0.001
2013	0.85 (0.73, 0.99)	0.03	0.61 (0.50, 0.74)	<0.001
2017	0.66 (0.57, 0.77)	<0.001	1.15 (0.98, 1.36)	0.1
2022	0.73 (0.62, 0.85)	<0.001	1.20 (1.01, 1.43)	0.04
**Maternal age**				
30+	Ref.	Ref.	Ref.	Ref.
20–29	1.05 (0.93, 1.18)	0.4	1.13 (1.00, 1.29)	0.06
<19	1.36 (1.10, 1.68)	0.004	1.12 (0.88, 1.44)	0.4
**Residence**				
Urban	Ref.	Ref.	Ref.	Ref.
Rural	0.92 (0.83, 1.03)	0.1	1.00 (0.88, 1.14)	1.0
**Education**				
Secondary and higher	Ref.	Ref.	Ref.	Ref.
Primary	1.20 (1.06, 1.36)	0.004	1.28 (1.12, 1.48)	<0.001
Incomplete primary	1.67 (1.40, 1.99)	<0.001	2.66 (2.23, 3.18)	<0.001
No education	1.85 (1.20, 2.85)	0.006	7.45 (5.37, 10.33)	<0.001
**Wealth index**				
Richest	Ref.	Ref.	Ref.	Ref.
Richer	1.07 (0.88, 1.31)	0.5	1.36 (1.00, 1.86)	0.05
Middle	1.00 (0.82, 1.22)	1.0	1.76 (1.31, 2.36)	<0.001
Poorer	1.25 (1.03, 1.52)	0.02	2.08 (1.56, 2.77)	<0.001
Poorest	1.40 (1.14, 1.71)	0.001	3.97 (2.98, 5.28)	<0.001
**Employment**				
Work	Ref.	Ref.	Ref.	Ref.
Did not work	1.09 (0.98, 1.20)	0.1	1.13 (1.00, 1.27)	0.05
**Parity**				
1	Ref.	Ref.	Ref.	Ref.
2	1.09 (0.95, 1.26)	0.2	1.04 (0.87, 1.24)	0.7
3+	1.17 (1.01, 1.36)	0.03	1.32 (1.12, 1.57)	0.001

**PCV**				
**Maternal age**				
30+	Ref.	Ref.	Ref.	Ref.
20–29	1.15 (0.96, 1.38)	0.1	1.31 (1.14, 1.50)	<0.001
<19	1.66 (1.21, 2.28)	0.002	1.32 (1.02, 1.71)	0.04
**Residence**				
Urban	Ref.	Ref.	Ref.	Ref.
Rural	0.99 (0.84, 1.18)	0.9	0.77 (0.68, 0.88)	<0.001
**Education**				
Secondary and higher	Ref.	Ref.	Ref.	Ref.
Primary	1.12 (0.93, 1.35)	0.2	1.15 (0.99, 1.33)	0.07
Incomplete primary	1.61 (1.17, 2.20)	0.003	2.43 (1.92, 3.08)	<0.001
No education	0.76 (0.28, 2.08)	0.6	4.40 (2.59, 7.50)	<0.001
**Wealth index**				
Richest	Ref.	Ref.	Ref.	Ref.
Richer	0.98 (0.72, 1.33)	0.9	1.35 (1.06, 1.72)	0.02
Middle	0.93 (0.70, 1.25)	0.6	1.16 (0.92, 1.47)	0.2
Poorer	0.95 (0.71, 1.27)	0.7	1.17 (0.93, 1.48)	0.2
Poorest	1.16 (0.87, 1.56)	0.3	1.62 (1.28, 2.05)	<0.001
**Employment**				
Work	Ref.	Ref.	Ref.	Ref.
Did not work	1.19 (1.01, 1.39)	0.04	1.30 (1.15, 1.48)	<0.001
**Parity**				
1	Ref.	Ref.	Ref.	Ref.
2	1.05 (0.85, 1.30)	0.7	1.14 (0.97, 1.34)	0.1
3+	1.17 (0.94, 1.46)	0.2	1.26 (1.06, 1.49)	0.008
